# High‐molecular‐weight DNA extraction for long‐read sequencing of plant genomes: An optimization of standard methods

**DOI:** 10.1002/aps3.11528

**Published:** 2023-06-13

**Authors:** Myoungbo Kang, Andre Chanderbali, Seungyeon Lee, Douglas E. Soltis, Pamela S. Soltis, Sangtae Kim

**Affiliations:** ^1^ Department of Biotechnology Sungshin Women's University Seoul 01133 Republic of Korea; ^2^ Florida Museum of Natural History University of Florida Gainesville Florida 32611 USA; ^3^ Department of Biology University of Florida Gainesville Florida 32611 USA

**Keywords:** CTAB, DNA extraction, Femto Pulse system, high‐molecular‐weight DNA, nuclei extraction

## Abstract

**Premise:**

Developing an effective and easy‐to‐use high‐molecular‐weight (HMW) DNA extraction method is essential for genomic research, especially in the era of third‐generation sequencing. To efficiently use technologies capable of generating long‐read sequences, it is important to maximize both the length and purity of the extracted DNA; however, this is frequently difficult to achieve with plant samples.

**Methods and Results:**

We present a HMW DNA extraction method that combines (1) a nuclei extraction method followed by (2) a traditional cetyltrimethylammonium bromide (CTAB) DNA extraction method for plants with optimized extraction conditions that influence HMW DNA recovery. Our protocol produced DNA fragments (percentage of fragments >20 kbp) that were, on average, ca. five times longer than those obtained using a commercial kit, and contaminants were removed more effectively.

**Conclusions:**

This effective HMW DNA extraction protocol can be used as a standard protocol for a diverse array of taxa, which will enhance plant genomic research.

The successful application of third‐generation sequencing technologies for sequencing nuclear genomes requires high‐molecular‐weight (HMW) DNA in sufficient quantity and quality for library preparation and sequencing (Healey et al., 2014). These DNA requirements are often challenging for non‐model plant species and represent an important bottleneck for plant genome research; therefore, the development of an efficient HMW DNA extraction method is essential for the plant genomics community. Although several approaches have recently been provided for HMW DNA extraction from plants, they were only applied to a few taxa, required additional purification steps, or the essential factors influencing the process were not adequately discussed (Healey et al., 2014; Mayjonade et al., 2016; Li et al., 2020; Cai et al., 2021; Jones et al., 2021; Mavrodiev et al., 2021; Zerpa‐Catanho et al., 2021). Therefore, there is a need for an easy‐to‐use protocol that can produce HMW DNA from a wide range of plant taxa at a low cost.

In this study, we propose a HMW plant DNA extraction method that combines two classic protocols: (1) a nuclei extraction method (Green et al., 1987) and (2) a cetyltrimethylammonium bromide (CTAB) plant DNA extraction method (Doyle and Doyle, 1987), with modifications. The nuclear extraction step reduces the ratio of organelle genomes in the extracted DNA (Hanania et al., 2004). The CTAB method has been modified in our protocol to solve the problems associated with phenolics and polysaccharides: polyvinylpyrrolidone (PVP) was added to isolate genomic DNA, as suggested by Healey et al. (2014). To more efficiently meet the needs of genome sequencing, our combined protocol includes (1) improvements to optimize time and reagent requirements and (2) suggestions of favorable conditions for factors influencing the results (number of pipetting steps, grinding time in liquid nitrogen, and centrifugation force in *g*). A combination of these two classic protocols has already been proposed for high‐quality DNA extraction from *Vitis vinifera* L. (Hanania et al., 2004), but not with regard to HMW DNA and applicability in other taxa. Similarly, a method combining the nuclear isolation process and sodium dodecyl sulfate (SDS)‐based DNA extraction protocol has recently been proposed for HMW DNA extraction (Zerpa‐Catanho et al., 2021); however, its effectiveness has only been confirmed in a few plant taxa (six genera in three families), and it requires an extra purification step (QIAGEN Genomic Tip 20/G columns; QIAGEN, Hilden, Germany). By contrast, we have assessed the broad applicability of our protocol in species representing 18 orders of flowering plants from all major angiosperm lineages (Angiosperm Phylogeny Group, 2016), as well as a gymnosperm, *Pinus* L.

To confirm the effectiveness of our HMW DNA extraction method, we compared the results with those obtained using a commercial plant DNA extraction kit. The DNA length distributions and purity were evaluated as validation criteria for comparing the two methods. We also discuss factors influencing the results, such as the number of pipetting steps, grinding time in liquid nitrogen, and centrifugation force in *g*.

## METHODS

### HMW DNA extraction method

We sampled leaves of species from each of 18 major angiosperm orders and one gymnosperm to test the taxon‐specific efficiency of our protocol. For details of all samples used in this study, see Appendix 1. Reagents, recipes, and a stepwise protocol are provided in Appendix 2. Our HMW DNA extraction protocol consists of three major steps: (1) grinding and nuclei isolation, (2) nuclear DNA extraction using CTAB buffer, and (3) RNase A and proteinase K treatment. We started with 2 g of tissue (preferably fresh, young leaves) and used a vacuum‐aided cell strainer (40 μm and 100 μm; pluriSelect Life Science, Leipzig, Germany) to collect the nuclei suspension. We also conducted additional DNA extractions using the same samples from our HMW DNA extraction protocol. For this, we employed the Exgene Plant SV kit (GeneAll Biotechnology, Seoul, Republic of Korea), a commercial plant DNA extraction kit based on the DNA‐binding filter method. Following the instructions in the manufacturer's manual, we used 0.1 g of leaf tissue, which is the recommended amount for fresh leaves.

#### Grinding and nuclei isolation

The protocol starts with 2 g of fresh, young leaves. We ground the leaves into a powder in liquid nitrogen (−80°C) and placed the powder in 20 mL of nuclei isolation buffer (IB). After 30 s of vortexing, we added Triton X‐100 (20 μL) and β‐mercaptoethanol (1.5 mL). This step should be conducted inside a fume hood as β‐mercaptoethanol is toxic. The samples were placed on ice for 10 min, and then the mixture was filtered through a 100‐µm cell strainer (pluriStrainer 100 µm; pluriSelect Life Science) seated in a 50‐mL conical tube to collect the nuclear suspension. During filtration, gently scraping plant material accumulated on the filter with the side of a 1000‐µL pipette tip may facilitate a smoother filtration. The filtering step was repeated with a 40‐µm cell strainer (pluriStrainer 40 µm; pluriSelect Life Science), and Triton X‐100 (200 µL) was added to the obtained nuclear suspension. This process lyses the cell and organellar membranes but not the nuclear membrane (Peterson et al., 1997). As a non‐ionic detergent, Triton X‐100 facilitates the release of nuclei from cells and prevents nuclei from clumping (Loureiro et al., 2007). To pellet the nuclei, the samples were centrifuged, and the supernatant was discarded. Centrifugation for 10 min at 3000 × *g* (4°C) is recommended to prevent fragmenting long DNA molecules (see Results).

#### Nuclear DNA extraction using CTAB buffer

The nuclei pellet was resuspended in 5 mL of Carlson Lysis Buffer (Carlson et al., 1991). Adding β‐mercaptoethanol (12.5 µL) denatures globular proteins to make them insoluble in water (Jadhav et al., 2015). An incomplete resuspension can reduce yield; thus, we incubated the samples at 65°C for a minimum of 15 min for efficient resuspension. If the pellet still does not suspend, crushing the pellet with a pipette tip might be helpful. For easy handling, we transferred the suspended nuclei pellet to a 15‐mL tube instead of proceeding with the 50‐mL tube. We added 5 mL (equal volume) of chloroform:isoamyl alcohol (24:1 [v/v]) to remove impurities. During this step, chloroform (CHCl_3_; a non‐polar 3‐hydrophobic solvent) dissolves non‐polar proteins and lipids to promote the partitioning of lipids and cellular debris into the organic phase. Isoamyl alcohol (C_5_H_12_O) prevents the emulsification of the solution (Jadhav et al., 2015). After centrifugation (3000 × *g* for 10 min at 4°C), the aqueous upper phase containing DNA was collected and transferred into a new tube, while the organic phase containing lipids, proteins, and other impurities was discarded. The separation of a pure aqueous phase is critical for the purity of the end product, and we recommend collecting just four‐fifths of the upper liquid volume to avoid including any cellular debris. Adding the proper ratio of sodium acetate (NaOAc) and isopropanol to the acquired supernatant is essential for precipitating the DNA: for every 10 mL of supernatant, a 1/10 volume of 3 M NaOAc (1 mL) and the same volume (including NaOAc) of room‐temperature isopropanol (11 mL) are needed. It is essential to use room‐temperature isopropanol for this step; otherwise, both polysaccharides and DNA will precipitate (Shepherd and McLay, 2011). The precipitated DNA was separated from other solvents through centrifugation (3000 × *g* for 10 min at 4°C), and the resulting DNA pellet was washed with 70% cold ethanol, recentrifuged (3000 × *g* for 10 min at 4°C), and thoroughly dried. We recommend rapidly drying samples using room‐temperature air blown by a hair dryer.

#### RNase A and proteinase K treatment

The DNA pellet was dissolved in 2 mL Tris‐EDTA (TE) buffer. To remove RNA and protein efficiently, which account for most of the impurities in extracted DNA, we treated the samples with RNase A (10 mg/mL) and proteinase K (>600 units/mL), respectively. For each treatment, the proper incubation time and enzyme activation temperature are important: 5 min at 37°C for RNase A and 15 min at 50°C for proteinase K. The enzymes used in each step are removed by a treatment with 2 mL of chloroform:isoamyl alcohol (24:1 [v/v]). After treatment with RNase A and proteinase K, the same precipitation procedure as for the CTAB extraction is followed. The resulting pellet is dissolved using an appropriate amount of deionized water (50–500 µL) according to the size of the pellet (recommended final concentration is ca. 200 ng/µL). If it is difficult to dissolve the pellet, we recommend incubating the tube at 50°C. If the pellet remains after incubation at 50°C, it is recommended to take only the dissolved aqueous layer after brief centrifugation.

#### Quality evaluation of extracted DNA

The quantity and purity (A_260_/A_280_ and A_260_/A_230_ ratios) of the extracted DNA were measured using a Qubit 4 Fluorometer (Thermo Fisher Scientific, Waltham, Massachusetts, USA) and a NanoDrop 2000 spectrophotometer (Thermo Fisher Scientific), respectively. The length distribution of the extracted DNA was evaluated using a Femto Pulse system (Agilent Technologies, Santa Clara, California, USA).

#### Optimization of conditions for HMW DNA recovery

We tested three factors influencing the results: (1) the number of pipetting steps, (2) the grinding time in liquid nitrogen, and (3) the centrifugation force in *g*. Three independent experiments were performed on different taxa in each case to evaluate each factor. First, we tested the impact of high *g* forces during centrifugation on DNA damage by comparing the setting in our protocol (3000 × *g*; control group) and a higher setting (5000 × *g*; experimental group). Second, the amount of grinding was compared. The control group was subjected to one minute of grinding (ensuring the sample was fully chilled before grinding began). The experimental group was subjected to an additional two minutes of grinding after adding extra liquid nitrogen. Third, we assessed whether high‐speed and multiple pipetting steps could potentially damage DNA. We conducted pumping at the maximum‐achievable speed 200 times in a tube using a P200 tip (experimental group) and compared the resulting DNA size distribution with the original DNA (control group).

## RESULTS

### DNA quantity, size, and purity measurements

**Figure 1:**
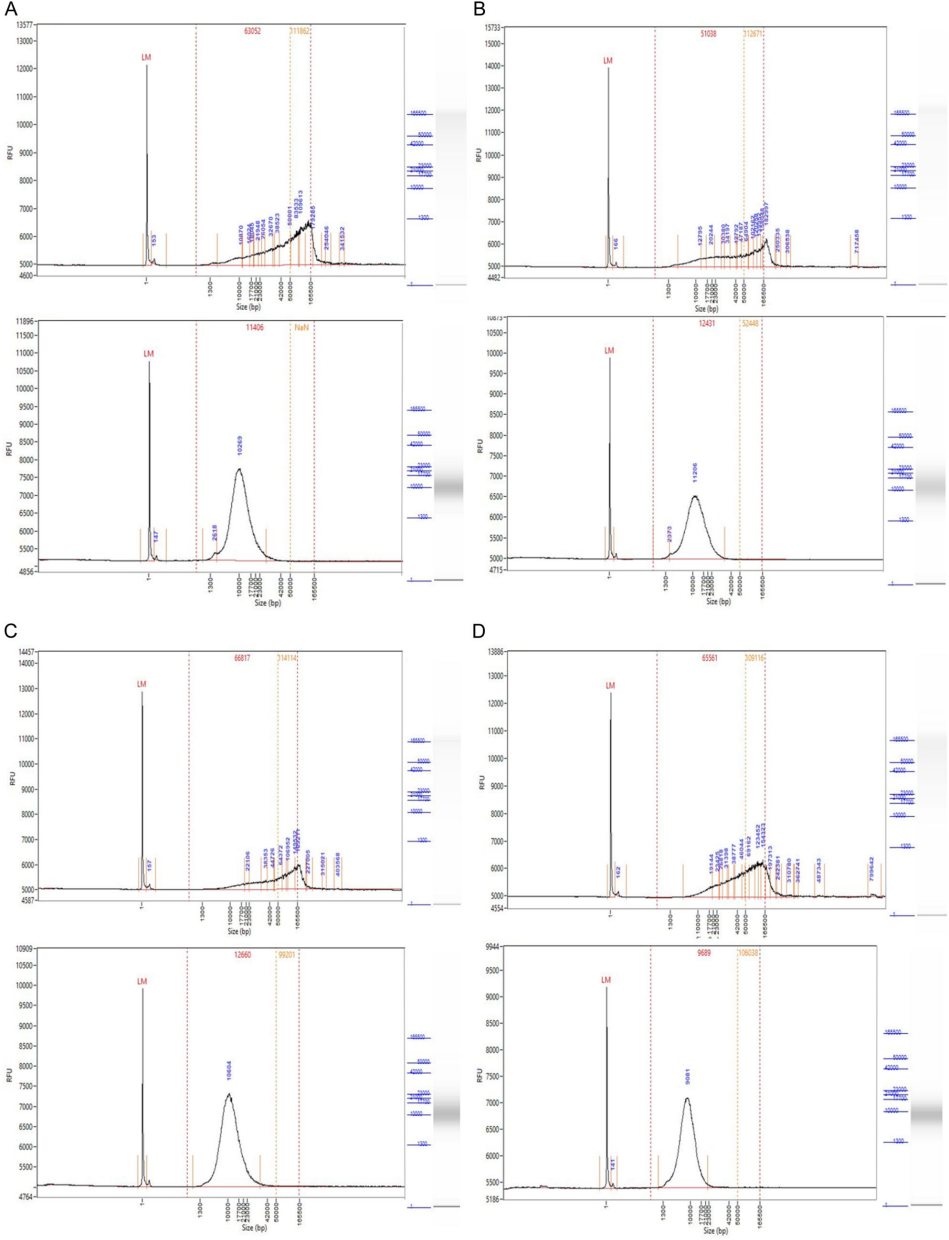
Comparison of the fragment‐length distributions of the extracted DNA estimated using the Femto Pulse system (Agilent Technologies, Santa Clara, California, USA) for selected examples: (A) *Spiraea prunifolia* var. *simpliciflora*, (B) *Alisma plantago‐aquatica* subsp. *orientale*, (C) *Epimedium koreanum*, and (D) *Lysimachia vulgaris* var. *davurica*. (A–D) The upper and lower graphs for each species represent the results of the HMW method and commercial kit, respectively. RFU, relative fluorescence units.

**Figure 2:**
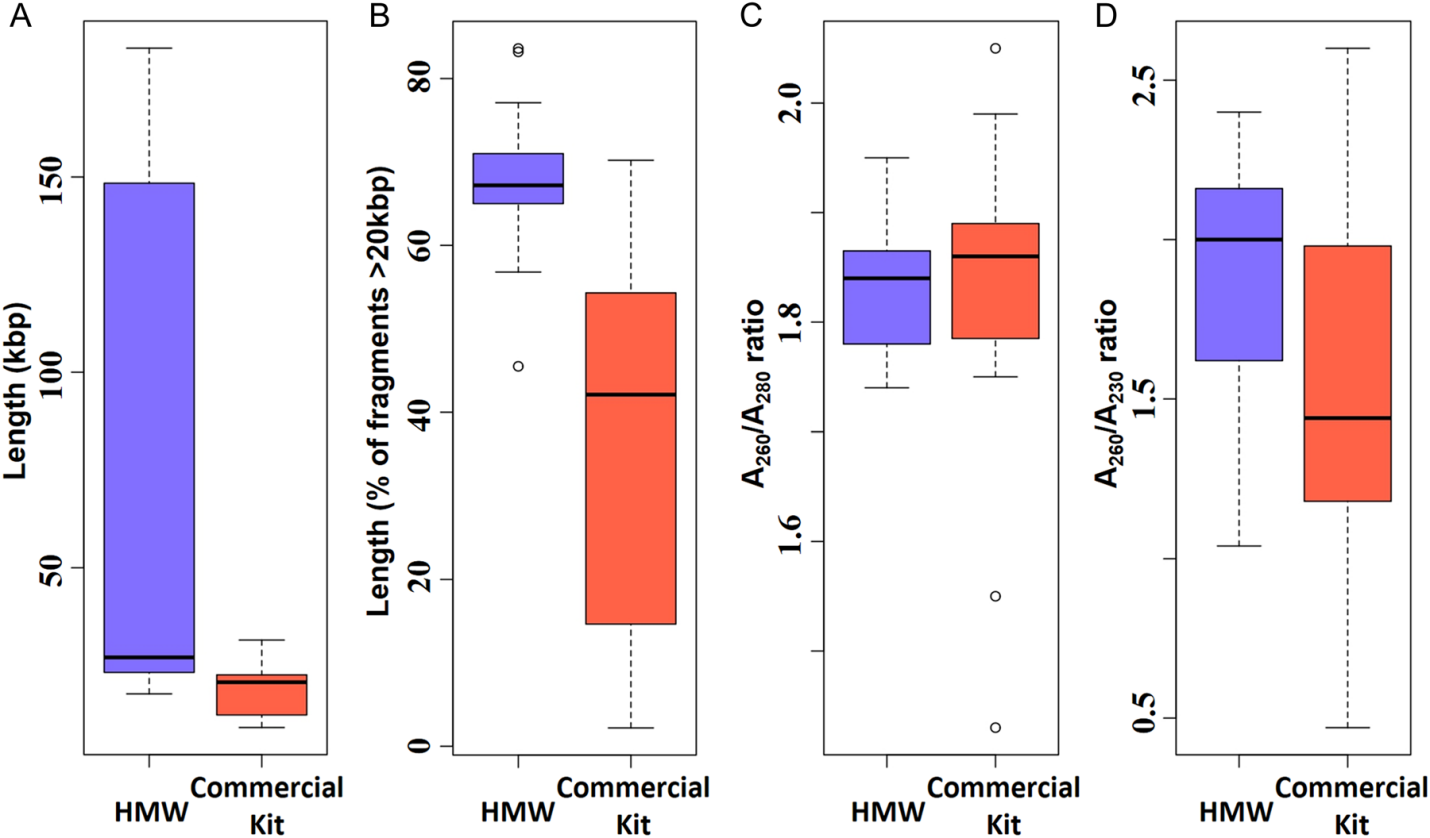
Comparison of the size and quality of DNA extracted using the two methods. (A, B) Size comparisons of (A) the highest peak and (B) the percentage of fragments >20 kbp. (C, D) Quality comparisons using (C) A_260_/A_280_ ratio and (D) A_260_/A_230_ ratio. The bold horizontal line in the middle of the box plot is the median value, and the lower and upper boundaries indicate the 25th and 75th percentiles, respectively.

Usually, the quantity of the end DNA product per extraction is enough to generate 4–5 libraries (8–15 µg) for long DNA sequencing with MinION or GridION (Oxford Nanopore Technologies, Oxford, United Kingdom), based on the library construction protocol (Ligation Sequencing Kit). The measurements obtained through the Femto Pulse system (peak height and percentages of fragments >20 kbp in the fragment‐length distribution graph) confirm that our protocol successfully produced DNA fragments an average of five times longer than those generated using the commercial kit (Table 1, Figure 1), although the results of our standard HMW DNA extraction protocol showed different patterns depending on the taxon (Figure 2A, B). With our protocol, the taxon with the highest portion of >20‐kbp fragments was *Chloranthus fortunei* Solms (Chloranthales; 83.6%), and the longest peak of DNA fragment distribution was obtained from *Alisma plantago‐aquatica* subsp. *orientale* (Sam.) Sam. (Alismatales; 183.0 kbp) (Table 1). In the most efficient instance, our protocol yielded 35 times more DNA fragments over 20 kbp (77.1%) in *Lysimachia davurica* Ledeb. (Ericales) than the commercial kit, for which only 2.2% of fragments were greater than 20 kbp.

**Table 1:** A comparison between our HMW DNA extraction method and a commercial kit. Fragment lengths were estimated using the Femto Pulse system.

Taxon	HMW method		Commercial kit		Ratio (a)/(b) × 100 (%)
	Peak (kbp)	% of >20 kbp (a)	Peak (kbp)	% of >20 kbp (b)	
*Platycladus orientalis*	21.57	58.8%	17.70	32.4%	181.5%
*Nymphaea tetragona* var. *minima*	22.10	59.7%	14.04	14.4%	414.6%
*Chloranthus fortunei*	38.21	83.6%	26.87	54.2%	154.2%
*Asarum sieboldii*	22.74	69.1%	28.81	66.5%	104.0%
*Alisma plantago‐aquatica* subsp. *orientale*	183.00	56.8%	11.21	10.8%	525.9%
*Hemerocallis fulva*	24.80	66.2%	31.48	70.2%	94.3%
*Carex breviculmis*	107.36	83.2%	22.19	52.4%	159.8%
*Epimedium koreanum*	169.21	67.2%	10.60	10.2%	658.8%
*Euonymus alatus*	27.04	67.0%	22.96	58.1%	115.3%
*Viola collina*	142.52	75.1%	10.69	14.9%	504.0%
*Spiraea prunifolia* var. *simpliciflora*	165.50	70.1%	10.27	7.2%	973.6%
*Pelargonium inquinans*	22.45	65.2%	21.20	55.7%	117.1%
*Aesculus turbinata*	24.00	66.1%	15.38	26.1%	253.3%
*Lysimachia davurica*	154.32	77.1%	9.08	2.2%	3504.5%
*Isodon inflexus*	23.88	71.9%	21.05	49.1%	145.5%
*Ipomoea nil*	23.65	68.4%	25.78	42.1%	162.5%
*Adenophora erecta*	132.21	67.2%	13.36	19.1%	351.8%
*Cicuta virosa*	17.70	45.5%	21.05	54.4%	83.6%
*Sambucus williamsii*	157.43	64.8%	20.67	44.0%	147.2%
Average	77.88 ± 64.48	67.53% ± 0.09%	18.65 ± 6.67	36.00% ± 0.21%	455.34% ± 7.55%

The quality of DNA extracted using the HMW method was superior to that obtained using the kit method in most samples. In the context of next‐generation sequencing, high‐quality DNA is characterized as predominantly HMW with an A_260_/A_280_ ratio over 1.8 and without contaminating substances, such as polysaccharides or phenolics (Kasem et al., 2008; Desjardins and Conklin, 2010). With both methods, the A_260_/A_280_ absorbance ratio, which measures protein contamination, showed similar results with low contamination (both averaged 1.83); however, our standard protocol more effectively removed carbohydrates and organic solvents (average A_260_/A_230_ ratio = 1.88) than the commercial kit (average A_260_/A_230_ ratio = 1.49) (Table 2; Figure 2C, D). Generally, A_260_/A_230_ values between 1.8–2.2 indicate DNA is free of carbohydrates and organic solvents (Kasem et al., 2008; Desjardins and Conklin, 2010).

**Table 2:** A comparison between our HMW DNA extraction method and a commercial kit. DNA purity was evaluated using a NanoDrop.

Taxa	HMW method		Commercial kit	
	A_260_/A_280_ ratio	A_260_/A_230_ ratio	A_260_/A_280_ ratio	A_260_/A_230_ ratio
*Platycladus orientalis*	1.77	1.32	1.55	0.56
*Nymphaea tetragona* var. *minima*	1.88	2.00	1.84	1.72
*Chloranthus fortunei*	1.87	1.97	1.89	1.44
*Asarum sieboldii*	1.88	1.46	1.86	1.29
*Alisma plantago‐aquatica* subsp. *orientale*	1.83	2.11	1.79	1.53
*Hemerocallis fulva*	1.79	2.33	1.79	2.60
*Carex breviculmis*	1.85	2.32	1.88	2.23
*Epimedium koreanum*	1.83	1.92	1.89	1.29
*Euonymus alatus*	1.86	2.19	1.75	0.92
*Viola collina*	1.94	2.40	1.86	2.17
*Spiraea prunifolia* var. *simpliciflora*	1.86	2.13	1.94	1.26
*Pelargonium inquinans*	1.84	2.09	1.99	1.16
*Aesculus turbinata*	1.76	1.62	1.43	0.47
*Lysimachia davurica*	1.84	1.14	1.85	1.20
*Isodon inflexus*	1.74	1.04	1.98	0.63
*Ipomoea nil*	1.76	1.62	2.05	1.80
*Adenophora erecta*	1.77	2.10	1.78	2.16
*Cicuta virosa*	1.82	1.77	1.78	1.52
*Sambucus williamsii*	1.95	2.20	1.87	2.29
Average	1.83 ± 0.06	1.88 ± 0.40	1.83 ± 0.14	1.49 ± 0.60

To address the statistical difference between the results from our protocol and a commercial kit, we performed paired *t*‐tests on all pairs of DNA length and quality, with *P* < 0.05 considered significant. In the DNA length criteria, the peak height and percentages of fragments >20 kbp show significant differences (*P* = 0.002 and *P* = 1.66e‐05, respectively). Because the DNA extracts from our method and the commercial kit both showed excellent A_260_/A_280_ ratios (both averaged 1.83), their quality was not significantly different (*P* = 0.7769); however, the A_260_/A_230_ ratio was significantly different (*P* = 0.001), indicating that our protocol provided an advantage.

### Factors influencing the results

#### Pipetting: avoid fast and frequent pipetting

Some long DNA extraction protocols suggest pipetting as little as possible or using a wide‐bore tip to avoid shearing (Zerpa‐Catanho et al., 2021). We confirmed that high‐speed repeated pipetting damages DNA. For samples extracted based on our protocol (control group), 17.0% of the DNA fragments were >50 kbp in length, with the peak being 76.46 kbp. In contrast with the control, the sample subjected to repeated pipetting (200 times) at high speed (experimental group) yielded just 13.4% of the fragments >50 kbp, with the peak being 49.93 kbp (Appendix 3A). High‐speed over‐pipetting, therefore, does affect HMW DNA extraction. The number of pipetting steps in our extraction protocol is fewer than 20, which is recommended to be performed gently with wide‐bore tips to reduce the likelihood of DNA shearing.

#### Grinding: avoid excessive grinding

Generally, it is important to grind samples as long as possible (at least 25 min or more [Circulomics, 2021], although in practice the grinding time is much shorter) in DNA extraction to transform the plant tissue into a powder. Excessive grinding can provide a yield advantage, but it can also shear the DNA. The DNA sizes of the samples ground to different degrees were compared with the Femto Pulse system, and we concluded that additional grinding for 2 min (experimental group) has a negative effect on DNA fragment length (Appendix 3B). One minute of grinding is optimal, and additional liquid nitrogen is not needed.

#### Centrifugation: avoid high speeds

Centrifugation causes DNA molecules to collide, resulting in their molecular structure being subjected to high shearing forces (Peterson et al., 2012). Our results showed that the DNAs of the experimental group (centrifuged at 5000 × *g*) are more fragmented than the control group (centrifuged at 3000 × *g*), with measured DNA peaks (indicating HMW DNA) of 88.73 kbp and 146.94 kbp, respectively, yielding 30.0% and 39.7% of DNA fragments >50 kbp, respectively (Appendix 3C). Given this difference in producing very long fragments (e.g., >50 kbp or more), high‐speed centrifugation over 3000 × *g* is not recommended.

## DISCUSSION

Here, we focused on optimizing and standardizing a HMW DNA extraction protocol for various plant taxa using economical techniques. We confirmed that our protocol successfully produced HMW DNA from various taxa in most cases; however, we expect that the experimental results will differ depending on the taxa investigated because each species has a different polysaccharide or phenolic content. Although not all species yielded good results using our protocol, we nevertheless confirmed that our protocol yielded DNA superior to the commercial kit in terms of length and purity, with statistically significant results.

To evaluate the results of various HMW DNA extraction methods, it is important to select an appropriate method and instrument with which the results can be compared. A common method to evaluate the length of the extracted DNA is a visualization of the position and brightness of DNA bands using electrophoresis through a low‐concentration agarose medium (typically 0.7%) containing ethidium bromide. Alternatively, more efficient electrophoresis can be performed using a pulse‐field power supply (e.g., Pippin Pulse system; Sage Science, Beverly, Massachusetts, USA). We tried pulse‐field electrophoresis to check the quality of HMW DNA at the initial stage of our study; however, we confirmed that the result (the brightest position of a smeared DNA band) varied depending on the amount of loaded DNA (Appendix 4). As the quantity of DNA loaded in the agarose gel for electrophoresis is increased, the brightest position of the DNA band is shifted to a higher position (a position of higher molecular weight); that is, the quantity of DNA and the brightest position of the DNA band are positively correlated. Special attention is therefore needed to ensure that the same quantity of DNA is used for each sample when evaluating DNA length using pulse‐field electrophoresis. Several automated electrophoresis techniques with fluorescence dye have been proposed for DNA length analysis to improve the unstable ethidium bromide visualization in normal electrophoresis (including pulse‐field). Although the TapeStation (Agilent Technologies) and the Fragment Analyzer (Agilent Technologies) are frequently used for size evaluations of extracted DNA fragments, they are not sensitive enough to separate HMW DNA (>60 kbp is not recommended in either instrument; Agilent Technologies, 2020a). Remarkably, the latter was used in a study of the development of a HMW DNA extraction protocol (Zerpa‐Catanho et al., 2021). By contrast, the Femto Pulse system is the automated pulsed‐field instrument designed for the purpose of analyzing HMW DNA. An automated pulsed‐field power supply in the Femto Pulse system allows the separation of DNA up to 165 kbp (Agilent Technologies, 2020b).

## CONCLUSIONS

The protocol introduced here can be used to efficiently extract HMW DNA using standard laboratory equipment (an average peak of 77.88 kbp and an average of 67.53% of fragments >20 kbp). Given its success with diverse flowering plant species and one gymnosperm, we hope our method will contribute to plant genome studies as a broadly applicable protocol for poorly studied taxa. Additional investigations comparing DNA length, purity, and extraction cost between our protocol and commercial HMW DNA extraction kits will provide a more comprehensive understanding of the benefits of our approach.

## AUTHOR CONTRIBUTIONS

S.K. and A.C. developed the experimental protocol. M.K. and S.K. performed all experiments and analyses. S.K. and M.K. wrote the preliminary manuscript draft, and all authors revised and approved the manuscript before submission. All authors approved the final version of the manuscript.

## Figures and Tables

**Figure A1:**
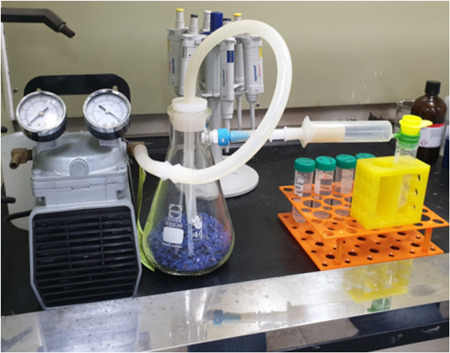
The setup of the vacuum‐aid filtration including a liquid‐overflow trap filled with silica gel. Using this setup can shorten extraction times.

## Data Availability

The taxonomic locations, vouchers, herbarium, and collection sites of all species used in this study are provided in Appendix 1.

## Appendix 1 Voucher information used in this study.

**Table:**

Higher classification^a^	Order	Species	Voucher (Herbarium and herbarium specimen number)^b^	Collection site
Gymnosperms				
	Pinales	*Platycladus orientalis* (L.) Franco	*M. Kang 0004* (SWU0036938)	37°37′54.87″N, 127°01′36.05″E
Angiosperm				
ANA grade				
	Nymphaeales	*Nymphaea tetragona* var. *minima* (Nakai) W. Lee	*M. Kang 0010* (SWU0053124)	37°37′54.93″N, 127°01′36.48″E
Unplaced	Chloranthales	*Chloranthus fortunei* Solms	*M. Kang 0003* (SWU0036937)	37°37′54.50″N, 127°01′35.97″E
Magnoliids				
	Piperales	*Asarum sieboldii* Miq.	*M. Kang 0001* (SWU0036935)	37°37′54.59″N, 127°01′36.24″E
Monocots				
	Alismatales	*Alisma plantago‐aquatica* subsp. *orientale* (Sam.) Sam.	*M. Kang 0011* (SWU0053125)	37°37′52.79″N, 127°01′34.56″E
	Asparagales	*Hemerocallis fulva* (L.) L.	*M. Kang 0005* (SWU0053111)	37°37′54.07″N, 127°01′36.43″E
Commelinids				
	Poales	*Carex breviculmis* R. Br.	*M. Kang 0026* (SWU0053782)	37°37′54.87″N, 127°01′36.05″E
Eudicots	Ranunculales	*Epimedium koreanum* Nakai	*M. Kang 0012* (SWU0053126)	37°37′55.06″N, 127°01′36.13″E
Core eudicots				
Rosids				
Fabids				
	Celastrales	*Euonymus alatus* (Thunb.) Siebold	*S. Kim 2021‐337* (SWU0053123)	37°37′49.07″N, 127°01′32.46″E
	Malpighiales	*Viola collina* Besser	*M. Kang 0023* (SWU0053779)	37°37′54.87″N, 127°01′36.05″E
	Rosales	*Spiraea prunifolia* var. *simpliciflora* (Nakai) Nakai	*M. Kang 0013* (SWU0053127)	37°37′55.02″N, 127°01′35.80″E
Malvids				
	Geraniales	*Pelargonium inquinans* (L.) L'Hér.	*M. Kang 0007* (SWU0053114)	37°37′53.31″N, 127°01′36.88″E
	Sapindales	*Aesculus turbinata* Blume	*M. Kang 0009* (SWU0053121)	37°37′54.07″N, 127°01′36.43″E
Asterids				
	Ericales	*Lysimachia davurica* Ledeb.	*M. Kang 0014* (SWU0053128)	37°37′54.91″N, 127°01′35.89″E
Lamiids				
	Lamiales	*Isodon inflexus* Kudô	*M. Kang 0002* (SWU0036936)	37°37′55.06″N, 127°01′36.06″E
	Solanales	*Ipomoea nil* (L.) Roth	*S. Kim 2021‐329* (SWU0053110)	37°38′26.06″N, 127°02′00.08″E
Campanulids				
	Asterales	*Adenophora erecta* S. Lee, Joongku Lee & S. Kim	*M. Kang 0006* (SWU0053113)	37°37′53.95″N, 127°01′35.40″E
	Apiales	*Cicuta virosa* L.	*S. Kim 2021‐331* (SWU0053116)	37°42′26.70″N, 128°36′53.56″E
	Dipsacales	*Sambucus williamsii* Hance	*M. Kang 0025* (SWU0053781)	37°37′54.87″N, 127°01′36.05″E

^a^APG IV (APG, 2016).

^b^Vouchers deposited at the herbarium of Sungshin University (SWU).

## Appendix 2 An optimized protocol for high‐molecular‐weight (HMW) DNA extraction in plant genomic studies.

**Note**: This protocol starts with 2 g of fresh, young leaves. Usually, the end product of one extraction process is sufficient to generate 4–5 libraries for sequencing with MinION or GridION (Oxford Nanopore Technologies, Oxford, United Kingdom).

### I. Preparation of solutions

1. **Preparation of nuclei isolation buffer (IB) (for 10 reactions)**
  - For 200 mL of nuclei IB, dissolve the following in ca. 100 mL of water:
    3 mL Tris‐HCl (1 M stock, pH 9.5; final concentration: 15 mM)
    4 mL EDTA (0.5 M stock; final concentration: 10 mM)
    1.94 g KCl (final concentration: 130 mM)
    0.8 mL NaCl (5 M stock; final concentration: 20 mM)
  - Gradually add 16 g of polyvinylpyrrolidone (PVP)‐10 while rapidly stirring the solution with a magnetic stir bar.
  - Use water to increase the volume to 200 mL.
  - Add 0.05 g of spermine and 0.07 g of spermidine. Store IB at 4°C.
  - Prepare 20 µL of Triton X‐100 and 1.5 mL of β‐mercaptoethanol, to be added after mixing the IB with the ground tissue (final concentrations of 0.1% and 7.5%, respectively; this constitutes IBTB).
    **Note**: Store at 4°C until use, or for a maximum of two weeks.
2. **Preparation of Carlson Lysis Buffer (Carlson et al.**, 1991 **)**
  - Carlson Lysis Buffer = 2× cetyltrimethylammonium bromide (CTAB) buffer + 1% polyethylene glycol (PEG) 6000
  - For 100 mL of Carlson Lysis Buffer:
  10 mL Tris‐HCl (1 M stock, pH 9.5; final concentration: 100 mM)
  4 mL EDTA (0.5 M stock; final concentration: 20 mM)
  8.2 g NaCl (final concentration: 1.4 M)
  2 g CTAB (final concentration: 2%)
  1 g PEG (final concentration: 1%)
  **Note**: Store at room temperature until use, or for up to two weeks.

3. **Tris‐EDTA buffer (TE) (1×)**

- TE buffer = 10 mM Tris‐HCl (pH 8.0) + 1 mM EDTA
  **Note**: Store at 4°C until use.

### II. Grinding and nuclei isolation (modified from Hanania et al., 2004)

1. Chill mortar and pestle at −80°C before beginning the extraction procedure. Grind 2 g of fresh, young leaves in liquid nitrogen for 1 min.
  **Note**: Ensuring the sample is fully chilled before grinding.
2. Add 2 g of ground leaf powder to 20 mL of IB in a 50‐mL conical tube and mix by inverting.
  **Note**: Over‐grinding negatively affects the extraction of HMW DNA. Grinding for 1 min is fine; additional grinding with extra liquid nitrogen is not needed.
  **Note**: Increase the sample amount for succulent plants, and increase the volume of IB when the mixture becomes viscous.
3. Immediately add 20 µL of Triton X‐100 and 1.5 mL of β‐mercaptoethanol and mix by inverting.
4. Keep on ice for 10 min.
  **Note**: This step should be conducted inside a fume hood because the IBTB contains β‐mercaptoethanol, which is toxic.
5. Filter the mixture through a vacuum‐aid cell strainer (pore size: 100 μm) seated on a 50‐mL conical tube to collect the nuclei suspension (Figure A1).
  **Note**: To aid filtration, gently scrape away plant tissue from the filter with the top of a 1000‐µL (blue) pipette tip. The filtrate should be light green.
6. Repeat the filtering step with a 40‐μm pore cell strainer.
7. Add 200 µL Triton X‐100 to the nuclei suspension.
  **Note**: This step lyses cell and organellar membranes, but not the nuclear membrane.
8. To pellet the nuclei, centrifuge for 10 min at 3000 × *g* at 4°C.
9. Discard the supernatant.

### III. Nuclear DNA extraction using CTAB buffer (modified from Doyle and Doyle, 1987)

1. Add 5 mL of Carlson Lysis Buffer and 12.5 µL β‐mercaptoethanol to the tube and resuspend the nuclei pellet with brief tapping.
  **Note**: Incomplete resuspension could reduce the yield as many nuclei will not have been lysed by CTAB. Briefly pipetting the pellet with an end‐cut 1000‐µL pipette tip and gentle vortexing may aid resuspension.
2. Incubate at 65°C for 15 min (maximum 2 h).
  **Note**: If the pellet is not completely resuspended after incubation, a brief centrifugation (3000 × *g* for 5 min) followed by only the use of the supernatant will help speed up processing.
3. Transfer the suspended nuclei pellet to a 15‐mL polypropylene tube and add an equal volume (5 mL) of chloroform:isoamyl alcohol (24:1 [v/v]) solution.
4. Invert several times to mix.
5. Centrifuge (3000 × g) for 10 min at 4°C.
6. Transfer the aqueous upper phase to a new tube using a P1000 pipette.
  **Note**: Take only 80% of the supernatant to avoid the inclusion of cellular debris. Take care removing the supernatant as this step is highly correlated with the quality of extracted DNA.
  **Note**: If the supernatant is viscous, slow pipetting will help avoid sucking up the plant tissue.
7. Repeat steps 3–6 (optional but highly recommended).
8. Add a 1/10 volume of 3 M sodium acetate (NaOAc), mix gently, add the same volume of isopropanol (room temperature), and gently invert several times.
  **Note**: For 4.5 mL of supernatant, add 0.45 mL of 3 M NaOAc and 4.95 mL of isopropanol.
9. Precipitate at –20°C for more than 1 h.
  **Note**: If precipitates are visible, moving to the next step is possible for faster processing. For highly viscous extracts, cold treatment makes the extract more viscous and more difficult to work with.
10. Centrifuge (3000 × *g*) for 10 min at 4°C.
11. Discard supernatant.
12. Wash pellets with 70% cold ethanol (ca. 20 mL per tube).
13. Centrifuge (3000 × *g*) for 10 min at 4°C.
14. Discard supernatant.
  **Note**: Keep the tube inverted for 1 min, and wipe out the tube wall with a Kimwipe.
15. Dry the pellet completely.
  **Note**: This step is very important for the quality of the DNA. The smell of alcohol is a good indicator of incomplete drying.

### IV. RNase A and proteinase K treatment

1. Dissolve the pellet with 2 mL of TE buffer.
  **Note**: If the pellet is difficult to dissolve, incubate in a 50°C water bath for up to 10 min.
  **Note**: Gently crushing the pellet with a pipette tip might be helpful for faster resuspension, but never vortex the sample. If the pellet is not completely resuspended after incubation, a brief centrifugation (3000 × *g* for 5 min) followed by only the use of the supernatant will help speed up processing.
2. Add 20 µL (10 µL/mL) of RNase A (10 mg/mL conc.).
3. Incubate at 37°C for 5 min.
4. Add 20 µL (10 µL/mL) of proteinase K (>600 units/mL conc.).
5. Incubate at 50°C for 15 min.
6. Add an equal volume (2 mL) of chloroform:isoamyl alcohol (24:1 [v/v]).
7. Invert several times to mix.
8. Centrifuge (3000 × *g*) for 10 min at 4°C.
9. Transfer the aqueous upper phase to a new 15‐mL tube.
  **Note**: Taking only 90% of the supernatant is best to avoid the inclusion of cellular debris. This is highly correlated with the quality of the extracted DNA.
10. Repeat steps 6–9 (optional).
11. Add a 1/10 volume of 3 M NaOAc, mix gently, add an equal volume of room‐temperature isopropanol, and gently invert several times.
  **Note**: For 3.5 mL of supernatant, add 0.35 mL of 3 M NaOAc and add 3.85 mL of isopropanol.
12. Precipitate at −20°C for more than 1 h.
  **Note**: If aggregates are visible, moving to the next step is possible for faster processing.
13. Centrifuge (3000 × *g*) for 10 min at 4°C.
14. Discard supernatant.
15. Wash pellets with 70% cold ethanol (ca. 5 mL per tube).
16. Centrifuge (3000 × *g*) for 10 min at 4°C.
17. Discard supernatant.
  **Note**: Keep the tube inverted for 1 min, and wipe out the tube wall with a Kimwipe.
18. Dry the pellet completely.
  **Note**: This step is very important for the quality of DNA. The smell of alcohol is a good indicator of incomplete drying.
19. Add 50−500 µL of deionized water to each tube to dissolve the pellet.
  **Note**: If it is difficult to dissolve, incubate in a 50°C water bath for up to 10 min.
  **Note**: Crushing the pellet with a pipette tip might be helpful for faster resuspension, but never vortex the sample. If the pellet is not completely resuspended after incubation, a brief centrifugation (3000 × *g* for 5 min) followed by only the use of the supernatant will help speed up processing.

### V. DNA size and quality measurements

1. Check the quality (A_260_/A_280_ and A_260_/A_230_ ratios) and quantity of extracted DNA using a NanoDrop 2000 (Thermo Fisher Scientific, Waltham, Massachusetts, USA) and a Qubit 4 Fluorometer (Thermo Fisher Scientific).
2. Check the length distribution of the DNA fragments using the Femto Pulse system (Agilent Technologies, Santa Clara, California, USA).

### VI. Special reagents and consumables

1. **Reagents**
  - PVP‐10: MilliporeSigma (Burlington, Massachusetts, USA) CAS 9003‐39‐8
  - Spermine: MilliporeSigma S2876
  - Spermidine: MilliporeSigma S2501
  - Triton X‐100: MilliporeSigma T8787
  - PEG 6000: MilliporeSigma 81260
  - RNase A: MilliporeSigma R6513
  - Proteinase K: MilliporeSigma P2308
2. **Consumables**
  - Vacuum‐aid cell strainer (100 μm): pluriSelect Life Science (pluriSelect Life Science, Leipzig, Germany) 43‐50100‐51 yellow 100 μm
  - Vacuum‐aid cell strainer (40 μm): pluriSelect Life Science 43‐50040‐51 blue 40 μm
  - Connector ring: pluriSelect Life Science 41‐50000‐03

## Appendix 3 Evaluation of factors influencing the DNA extraction process, using three taxa each as examples. Effects of (A) pipetting repeats (experimental group: additional 200 pipetting pumps with P200 tip), (B) degree of grinding (experimental group: additional 2 min of grinding with a second pour of liquid nitrogen), and (C) centrifugation force (control group: 3000 × *g*; experimental group: 5000 × *g*).

**Table:**

Example taxa for each factor	DNA fragment length			
	Peak (kbp)		% of fragments >50 kbp	
	Control group (a)	Experimental group (b)	Control group (a)	Experimental group (b)
**(A) Pipetting**				
*Chloranthus fortunei*	25.99	22.00	12.5%	7.7%
*Alisma plantago‐aquatica* subsp. *orientale*	165.50	91.6	26.1%	19.1%
*Scutellaria insignis*	37.89	36.18	12.3%	13.3%
Average ± standard deviation	76.46 ± 63.15	49.93 ± 30.03	17.0% ± 0.06%	13.4% ± 0.05%
Average (a) – average (b)	26.53 ± 41.04		3.6% ± 0.04%	
**(B) Grinding**				
*Chloranthus fortunei*	32.37	29.30	21.5%	0.0%
*Carex breviculmis*	107.36	88.95	34.7%	35.8%
*Viola collina*	142.52	147.49	46.8%	42.9%
Average ± standard deviation	94.08 ± 45.94	88.58 ± 48.25	34.3% ± 0.10%	26.2% ± 0.18%
Average (a) – average (b)	5.50 ± 11.88		8.10% ± 0.12%	
**(C) Centrifugation**				
*Chloranthus fortunei*	151.12	92.37	42.4%	31.1%
*Scutellaria salviifolia*	132.27	46.21	41.4%	27.7%
*Sambucus williamsii*	157.42	127.62	35.3%	31.4%
Average ± standard deviation	146.94 ± 10.69	88.73 ± 33.33	39.7% ± 0.03%	30.0% ± 0.02%
Average (a) – average (b)	58.20 ± 22.97		9.63% ± 0.04%	
